# Defence mechanisms: the role of physiology in current and future environmental protection paradigms

**DOI:** 10.1093/conphys/coy012

**Published:** 2018-03-13

**Authors:** Chris N Glover

**Affiliations:** 1 Faculty of Science and Technology and Athabasca River Basin Research Institute, Athabasca University, Canada; 2 Department of Biological Sciences, CW 405, Biological Sciences Bldg. University of Alberta Edmonton, Alberta, Canada T6G 2E9

**Keywords:** Adverse outcomes pathway, biotic ligand model, metabolism, risk assessment, toxicokinetics, toxicodynamics

## Abstract

Ecological risk assessments principally rely on simplified metrics of organismal sensitivity that do not consider mechanism or biological traits. As such, they are unable to adequately extrapolate from standard laboratory tests to real-world settings, and largely fail to account for the diversity of organisms and environmental variables that occur in natural environments. However, an understanding of how stressors influence organism health can compensate for these limitations. Mechanistic knowledge can be used to account for species differences in basal biological function and variability in environmental factors, including spatial and temporal changes in the chemical, physical and biological milieu. Consequently, physiological understanding of biological function, and how this is altered by stressor exposure, can facilitate proactive, predictive risk assessment. In this perspective article, existing frameworks that utilize physiological knowledge (e.g. biotic ligand models, adverse outcomes pathways and mechanistic effect models), are outlined, and specific examples of how mechanistic understanding has been used to predict risk are highlighted. Future research approaches and data needs for extending the incorporation of physiological information into ecological risk assessments are discussed. Although the review focuses on chemical toxicants in aquatic systems, physical and biological stressors and terrestrial environments are also briefly considered.

## Teaser

Integrating physiological mechanism into ecological risk assessment has multiple advantages, as demonstrated by approaches such as the biotic ligand model, adverse outcomes pathways and mechanistic effects models. However, there is significant additional scope for incorporating physiological data and principles into environmental protection paradigms.

## Introduction

Physiology provides the mechanism that underpins our understanding of ecology, evolution, health and disease. It also facilitates the translation of molecular and cellular responses of individual organisms, to changes observed at the population, community and ecosystem scale (Somero, 2000). As such there is significant crossover between physiology as a discipline, and the goals of conservation, particularly as they pertain to assessing threats to the environment. For example, ecological risk assessment seeks to determine whether a given chemical, physical (i.e. climatic, land-use or hydrological change) or biological (e.g. invasive species or disease) stressor will result in a negative ecological outcome. Historically, environmental risk has been largely determined by monitoring the toxicity of stressors to individual model species, in controlled laboratory settings, using crude metrics such as mortality (Maruya *et al.*, 2016). These data have then been extrapolated to discern levels of stressors that will have no, or only a minor, environmental impact. As such, risk assessments take individual-level impacts, which result from suborganismal perturbation, and seek to translate these effects to larger scales (Rohr *et al.*, 2016). It is, therefore, surprising that environmental risk assessment has traditionally eschewed physiological approaches that would appear to contribute directly to regulatory goals. For example, mechanistic knowledge of how a stressor impacts an organism facilitates predictive, and thus proactive, approaches to assessing risk; it allows an understanding of processes such as acclimation that could affect stressor impact; and it enables evaluation of risk under real-world scenarios where multiple stressors are simultaneously acting upon an organism. Hence, physiological knowledge can provide the basis for versatile and robust risk assessment tools that utilize an understanding of stressor impacts at an individual level, but which are applicable to the prediction of effects across biomes, across species, and in the presence of multiple stressors (Segner *et al.*, 2014). The current Perspective initially outlines traditional risk assessment paradigms, highlighting that these approaches generate values that have a limited mechanistic basis. It then provides a generic overview of more modern approaches that have the potential to incorporate mechanistic information, and predictive capacity, into risk assessment frameworks. Thereafter, selected case studies illustrating how mechanistic information has been utilized within the context of the stressor-organism nexus are outlined. Finally, opportunities for future physiologically based research efforts that could contribute significantly to an improved understanding of the drivers of toxicological impact are identified. Overall, the aim of this work is to offer insight into the regulatory environment from a physiological perspective, and to identify where physiological knowledge has, and could further, contribute to risk assessment approaches.

## Regulatory background

Most current risk assessment practices are designed with a focus on the stressor. In the case of anthropogenic toxicants, approaches seek to determine the ‘safe’ concentration of a given chemical, and give little consideration to the biological receptor (Maruya *et al.*, 2016; Burton, 2017). Traditional risk assessment is also characterized by its reductionist approach, condensing toxicological information down to a single value or set of values (Jager, 2016). This simplification is understandable given the ever-growing complexity of the chemical milieu, the variety of human-induced alterations in physicochemical properties of ecosystems, and the diverse nature of these ecosystems and the biota therein.

### Single end-point values

The standard approach to assessing the environmental risk of a stressor is laboratory toxicity testing (Chapman *et al.*, 1998). Taking a single species, toxicity tests over fixed time intervals are performed under standard conditions (e.g. temperature, water chemistry). The choice of species is often stipulated by a regulatory body, and is almost invariably a standard toxicological model. These are organisms that are amenable to laboratory culture, but which may hold little relevance to an environmental scenario (Segner and Baumann, 2015). These tests, conducted over acute or chronic timeframes, generate values that summarize the sensitivity of the species towards a stressor. When examining chemical toxicants, these values include the median lethal concentration (LC_50_), and the lowest tested concentration at which no (no observable adverse effect concentration; NOEC) or some (lowest observable adverse effect concentration; LOEC) effect is noted. Sometimes, rather than mortality, an alternative end-point may be used (median effect concentration; EC_50_), such as growth in assessments of algal toxicity, or reproduction in chronic assays. Measures of survival, growth and reproductive capacity are all derivations of the interaction of the stressor with organism, and thus reflect physiological impairment. However, these simple end-points do not consider at all the mechanism by which species survival/function has been compromised (Chapman *et al.*, 1998). As such there is no understanding of why end-point values may differ between species, life-stages or populations, and thus there is little predictive power in such metrics.

### Species sensitivity distributions

A more integrative tool for assessing risk is the species sensitivity distribution (SSD). Here, end-point values for multiple species are combined, a distribution is fitted to the data, and a hazard concentration is determined (e.g. a stressor concentration that protects 95% of tested species; Wheeler *et al.*, 2002). As such, SSDs contribute directly to risk assessment goals (e.g. protect 95% of the species, 95% of the time; van Straalen and van Rijn, 1998). While SSDs are clearly more robust than single end-point values alone (Hahn *et al.*, 2014), they suffer from similar criticisms. For example, studies are still performed in a relatively few species, and under standard test conditions. Furthermore, the outcomes of SSD analyses depend on the number of data points used to construct them, and the nature of the distribution fitted (Forbes and Calow, 2002). Some SSD approaches do normalize end-point values on the basis of bioavailability (van Sprang *et al.*, 2016), and as bioavailability is driven by organismal physiology, then such analyses do incorporate mechanistic understanding. However, while SSDs can show that differences in sensitivity between species exist, and can identify which of the test species are more sensitive, the mechanisms underlying these differences are not directly investigated.

## Shifting paradigms

**Figure 1:**
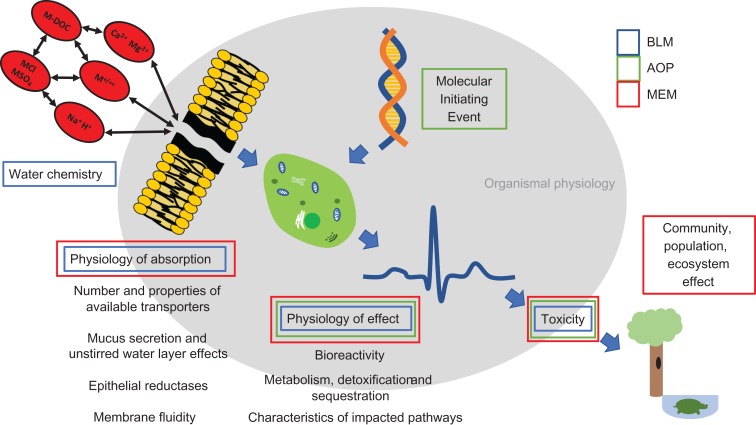
Diagrammatic representation of modelling frameworks (BLM, biotic ligand models; AOP, adverse outcome pathways; MEM, mechanistic effect models) incorporating physiological considerations for the purposes of risk assessment.

The weaknesses in simple end-point analyses and SSD approaches have been recognized for some time (Forbes and Calow, 2002). For example, a contributing factor to the perceived failure of pesticide risk assessments in tropical regions of Australia is that SSD methods resulted in trigger values significantly greater than concentrations at which sub-lethal effects may be induced (Holmes, 2014). This flaw would likely have been mitigated by a better understanding of biological mechanisms of pesticide impact. Consequently, driven by the need for risk assessment methods that integrate biological knowledge, and which facilitate predictive, proactive assessment, a number of mechanism-based tools that incorporate physiological measurements have been proposed/developed. These include biotic ligand models (BLM), adverse outcome pathways (AOP) and a variety of mechanistic effects models (MEM). While current BLMs are specific for chemical toxicants, AOP and MEM approaches are applicable to a broader range of stressors. The general framework of each of these tools, and their integration of physiological information, is outlined in Fig. 1, and discussed below.

### Biotic ligand model

The BLM approach is based on an understanding of the relationship between water chemistry, toxicant speciation, critical tissue burden, and mechanisms of toxicant uptake and effect (Di Toro *et al.*, 2001; Niyogi and Wood, 2004). The best developed BLMs are those applied to assessing metal toxicity in freshwaters. Based on physiological studies that show metal toxicants are mimics of essential ions, the BLM approach uses water chemistry to establish the amount of bioavailable metal (i.e. that which is ionic and thus capable of being absorbed through epithelial transporters; Bury *et al.*, 2003). This information is then coupled with experimental analysis of the relationship between bioavailability, the short-term (e.g. 3 h) accumulation of metal at a sensitive site (gills of fish, or whole bodies of small invertebrates), and longer-term (e.g. 96 h) toxic effect (e.g. mortality, inhibition of ion uptake), to determine whether the concentration of a metal in a given water body is likely to be harmful (Di Toro *et al.*, 2001). Consequently, the BLM approach relies absolutely on physiological understanding of uptake pathways and mechanism of toxicity. Importantly, because of its mechanistic underpinning, this is a model that has been shown to have relevance in different environmental matrices (e.g. saline waters, Arnold *et al.*, 2005; sediments, Di Toro *et al.*, 2005; soils, An *et al.*, 2012), and has been applied to the complex issue of stressor mixtures (Iwasaki *et al.*, 2015). Because of robust validation, and its capacity for predicting toxicity proactively, the BLM approach has been approved as an acceptable regulatory tool in many jurisdictions worldwide (Rüdel *et al.*, 2015).

There are, however, acknowledged weaknesses to the BLM approach. For example, it does not account easily for toxicants sourced from the diet (Niyogi and Wood, 2003). The diet is the major route of metal exposure, but metals absorbed by this pathway can have distinct bioavailability and bioreactivity owing to the presence of absorbable toxicant-organic ligands, thus distorting the relationship between body burden and toxicity. Current BLMs are also limited in that they do not account for acclimation, wherein the uptake route and/or the toxicologically sensitive pathway change in response to prolonged and/or prior exposure (Niyogi and Wood, 2003). One further important assumption of the BLM is that mechanisms of uptake and toxicity are conserved between life-stages and species. However, testing of these assumptions has focussed on a relatively narrow range of test species, a legacy of standard toxicity tests. Recent work, seeking to expand the BLM approach from standard North American fish species to key Southern Hemisphere fish, has shown that while the mechanisms of effects are generally conserved, there are important differences. For example, *Galaxias maculatus*, a widely distributed southern temperate fish species, has a scaleless skin surface which performs a number of physiological roles (Glover *et al.*, 2013), including ion transport (Harley, 2015). It has been proposed that the skin of these species may serve both as an alternative pathway of toxicant uptake, but also as a rescue pathway, taking on physiologically important functions that may be compromised at the gill of metal-exposed fish (Glover *et al.*, 2016; McRae *et al.*, 2016). This observation does, however, further serve to highlight the importance of physiological knowledge in risk assessment, particularly when applying guidelines across biomes.

It is also worth noting that although BLM approaches have been adopted by regulatory authorities worldwide, they are not always employed in the manner for which they were designed. The BLM approach was developed to assess acute toxicity, but is often used as a predictor of chronic toxicity (Niyogi and Wood, 2004). This is a practice that assumes mechanisms of toxicity, and the relationships between accumulation and effect, are independent of exposure duration. This assumption remains unverified for many toxicants (Niyogi and Wood, 2004). Furthermore, chronic BLMs are often developed by implying, rather than measuring, a toxicant burden at the sensitive site (Villavicencio *et al.*, 2011). If coupled with methodologies where the chronic toxicity itself is a calculation based on acute-to-chronic extrapolation (USEPA, 2007), then this has the potential to greatly diminish the mechanistic basis of the BLM approach.

### Adverse outcome pathway

**Figure 2:**
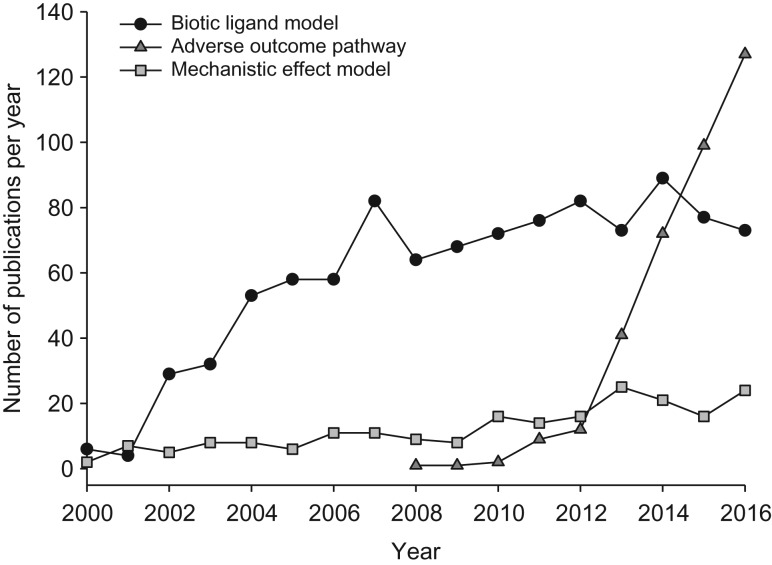
Growth in peer-reviewed journal publications incorporating physiological considerations for the purposes of risk assessment, from 2000 to 2016 (ISI Web of Science).

Another mechanistic approach to risk assessment is the AOP framework (Ankley *et al.*, 2010), which has received significant attention from the research community since its conception (Fig. 2). An AOP seeks to build mechanistic knowledge of how a stressor impacts the biology of an exposed organism. The theoretical framework of the AOP is that an initial perturbation of molecular function (termed a ‘molecular initiating event’), causes changes at a biochemical and cellular level, resulting in a physiological consequence (Ankley *et al.*, 2010). Theoretically, stressors that induce similar changes in molecular and/or biochemical responses, will have similar physiological and toxicological consequences. Thus, the identification of shared pathways of effect acts as a mechanism by which grouping of stressors by their mechanism of action can occur, simplifying risk assessment, and facilitating prediction of toxic impact. Furthermore, knowledge of the properties of impacted physiological pathways in receptor species will allow prediction of their sensitivity. Importantly, the AOP framework is of significant potential utility in solving many of the issues associated with predicting the toxicological impacts of stressor mixtures (Kienzler *et al.*, 2016). To date, AOPs have been postulated for a number of important environmental contaminants (e.g. nanoparticles, Muller *et al.*, 2015; metals, Brix *et al.*, 2017; endocrine disrupting chemicals, Song *et al.*, 2017).

The AOP approach shares many weaknesses with the BLM, including the assumption that the AOP for a given stressor in one species is likely to be applicable to others, and a limited scope to account for acclimation effects (Rohr *et al.*, 2016). Furthermore, altered function at a molecular level may not directly translate to meaningful change at an organismal level. For example, compensation of functions by alternate pathways may mean that changes in the expression of specific genes or proteins do not translate to physiological changes, and consequently may have limited value for environmental protection (Feder and Walser, 2005). Similarly, not all physiological changes that may be induced by exposure to a stressor will be relevant to measures of fitness, and even for those changes that are pertinent, there may be limited knowledge of the thresholds at which these changes result in ecologically relevant effects. It has also been suggested that the large number of molecular changes that may be induced by stressor exposure, may confound identification of biologically meaningful events, and lead to AOPs that do not truly reflect cause and effect pathways that facilitate prediction (Escher *et al.*, 2017). For this reason, top-down approaches, where an observed adverse outcome initiates the elucidation of causative mechanisms, may have more predictive value than bottom-up approaches, where known mechanisms of stressor effect are linked to adverse outcomes.

### Mechanistic effect model

This is an umbrella term that covers a range of different approaches that incorporate modelling to extrapolate effects at an organism level, to broader community, population and ecosystem settings (Forbes and Calow, 2012). These are usually frameworks that have been developed for ecological modelling, adapted to account for the presence of a stressor. As such they provide a means of relating traditional laboratory-based studies, to impacts at levels of organization of greatest relevance to risk assessors (Grimm and Martin, 2013). A key feature of MEMs is their ability to assess interspecific interactions. For example, MEM approaches show that when interspecies competition is accounted for in a system receiving a pulsed pesticide exposure, population recovery rates (based on physiological knowledge of reproductive parameters) could be as much as three-fold longer, compared to scenarios where competition effects are not considered (Kattwinkel and Liess, 2014).

The MEMs of perhaps the greatest physiological relevance, are toxicokinetic-toxicodynamic (TKTD) models (Ashauer and Escher, 2010). Simply stated, toxicokinetic processes are those through which the organism can affect the toxicant. These therefore include absorption, distribution, metabolism and excretion. As these are factors strongly controlled by organism physiology, then greater predictive power can be gained by the incorporation of physiologically based toxicokinetic (PB-TK) models, which account for the critical molecular, biochemical and physiological processes that impact kinetics (Grech *et al.*, 2017). Toxicodynamic processes are those wherein the toxicant impacts the organism, and therefore incorporate mechanisms of toxicity. When combined into TKTD or PB-TKTD models, factors such as exposure concentration, exposure duration, organism size, metabolic rate, molecular, cellular and whole organism physiological parameters can be used to predict toxicity. Importantly, these models are able to account for temporal variability in exposures, and factors such as organism acclimation to exposure over time (Ashauer and Escher, 2010). They are also easily adjusted to account for physiological differences in model parameters between species (Nyman *et al.*, 2014).

Mechanistic effects models can be very complex, a characteristic that can limit their utility in risk assessments (Hommen *et al.*, 2016). However, when complexity is eschewed for more simple approaches, the integrity of the models is often compromised (Rowland *et al.*, 2017). Furthermore, the strength and utility of a given model is dependent on both the quantity and quality of the data upon which it is based. Currently, the MEM approach is limited by insufficient knowledge of physiological parameters across receptor species (Ippolito *et al.*, 2012). Despite this, there are examples where MEMs have provided improved risk prediction where traditional single end-point values and laboratory lethal toxicity tests have failed. For example, the pesticide additive Dispersogen A has low toxicity in a standard chronic *Daphnia magna* toxicity test with reproduction as an end-point, but exposure to a predicted NOEC in a population growth test resulted in a near 20% decline in population (Hammers-Wirtz and Ratte, 2000). This effect was likely mediated by a decline in offspring quality, a factor unaccounted for in traditional testing protocols. However, an MEM approach incorporating multiple end-points was able to accurately predict the effects of Dispersogen A on population size (Gabsi *et al.*, 2014).

## Applications of physiological knowledge to risk assessment

There is a strong theoretical justification for incorporating mechanistic knowledge into risk assessment. Indeed, in practice there have been some significant advances towards this goal. Selected diverse case studies where physiological understanding has already made a contribution to predictive risk assessment are described below.

### Case study A—Silver toxicity to freshwater biota

Knowledge of how silver causes toxicity in aquatic animals is an excellent example of how mechanistic understanding can contribute directly to regulatory risk assessment. Waterborne silver is toxic to aquatic biota, but only in its ionic form (Ag^+^). The physiological basis for this observation is that ionic silver mimics sodium, and therefore gains access to the animal through sodium transport pathways (Wood, 2012). Other chemical species of silver are not bioavailable, and thus do not contribute to toxicity. This understanding revolutionized risk assessment for silver in the environment, helping to modify regulations based on total metal which were overly restrictive, to those that accounted for metal speciation, eventually leading to approaches such as the BLM (Adams *et al.*, 2002).

Once absorbed, the main mechanism of silver toxicity is the inhibition of ion transport, through impairment of the basolateral sodium pump, which maintains the electrochemical gradient that achieves ion regulation. As silver toxicity correlates with silver burden at sensitive sites (gill of fish, whole body of invertebrates; Wood, 2012), then it holds that toxicity will be strongly shaped by the characteristics of sodium uptake pathways. This partly explains the high sensitivity of freshwater animals to silver, as these species must have high rates of sodium uptake to compensate for sodium lost via diffusion from their more concentrated bodies to the surrounding environment. Furthermore, it also explains the finding that small freshwater animals are especially sensitive to silver toxicity (Grosell *et al.*, 2002). These species and life-stages have a high surface area to body volume ratio, and thus rely absolutely on effective sodium uptake to maintain homoeostasis.

Importantly, because of the underlying physiological basis of silver toxicity, models that predict sensitivity of silver can be applied across settings that differ in their water chemistry (i.e. salinity), by geochemical modelling or direct measurement of free silver ion (Nichols *et al.*, 2006). Furthermore, assuming that mechanisms of silver toxicity are conserved, then BLMs developed in model species can be parameterized to non-model animals, with knowledge of sodium uptake mechanisms, or even body size (Veltman *et al.*, 2014). Because of this utility, and the confidence associated with a robust understanding of underlying physiological mechanism, silver BLMs are important regulatory tools in a number of jurisdictions worldwide (Wood, 2012).

### Case study B—Climate change and flying insect distribution

Physiology can aid significantly in the prediction of how increasing global temperature trends are likely to impact upon species distributions (Evans *et al.*, 2015). For most environmental stressors there is only a limited understanding of their temporal variability. For example, factors such as improved industrial processes, remediation, and complex natural degradation pathways, means that chemical stressor concentrations fluctuate and are not easily predicted (Iqbal and Oberg, 2013). In the case of climate change, owing to the global nature of the problem, intervention will have only a limited impact on temperatures in the immediate future. Thus, while the predictions of the extent of warming differ, there is little doubt as to the general trend in this stressor over time, and its climatic impacts (Heikkinen *et al.*, 2006). Consequently, predictions of species responses to climate are likely to be of significant real-world value (Helmuth *et al.*, 2005).

Some of the most important studies utilizing physiological measures to enhance our understanding of biological responses to climate change have been performed in flying insects. For example, the increasingly early emergence of the butterfly *Heteronympha merope* in recent years has been linked to knowledge of the role of temperature in development processes in early life-stages of this species (Kearney *et al.*, 2010). Similarly, Buckley and colleagues (2011) incorporated species-specific knowledge of the relationship between temperature and developmental rate to fit a model to historical data of butterfly distribution. When applied to a later dataset, this model was a better predictor of butterfly distributions than a model which assumed butterfly development as a function of temperature was fixed between species (Buckley *et al.*, 2011), thus demonstrating the importance of specific physiological knowledge within climate change impact scenarios. In another example, assessment of critical thermal maxima (a physiological metric of thermal tolerance), predicted the population responses of different bee species to urban heat-island effects (Hamblin *et al.*, 2017). Finally, there is some evidence in flying insects that supports the Oxygen- and Capacity-Limited Thermal Tolerance (OCLTT) hypothesis, which suggests that oxygen acquisition is a limiting factor for animals as oxygen demand increases (e.g. as a function of elevated environmental temperature; Pörtner *et al.*, 2017). Specifically, it has been shown that anaerobic metabolites increase at thermal limits in the fruit fly (*Drosophila melanogaster*; Malmendal *et al.*, 2006) and an Antarctic midge (Michaud *et al.*, 2008), while hypoxia exposure reduces thermal tolerance in *Drosophila* (Lighton, 2007). The OCLTT is an appealing hypothesis as it strongly links physiological mechanisms (e.g. respiration, blood flow) to ecologically relevant effects (e.g. changes in animal distribution). The utility of physiological information for predicting species responses to a changing climate is not restricted just to terrestrial insects, but has value to other terrestrial organisms (Marin *et al.*, 2014; Mathewson *et al.*, 2017), and to aquatic biota (Martinez *et al.*, 2015).

### Pesticide toxicity and water permeability in aquatic insects

Owing to the relationship between uptake and toxicity for most chemical toxicants, measures of accumulation can offer insight into toxicity. In a study examining ten aquatic insect taxa exposed to the organophosphate pesticide chlorpyrifos, it was shown that aerially respiring insects accumulated less toxicant than aquatic breathers (Buchwalter *et al.*, 2002). Aerial respiration is problematic owing to the risk of desiccation, but this is compensated for by the higher oxygen content relative to water. These factors are both drivers facilitating a reduced relative size of the respiratory surface in air breathing taxa (Maina, 2002). Thus, when aerially respiring animals are exposed to membrane-permeable toxicants such as chlorpyrifos, there is relatively less surface area across which absorption can take place, resulting in a relatively lower body burden, and thus reduced toxic impact. As such, knowledge of respiration mode is a strong predictor of pesticide toxicity in aquatic insects. However, it is important to note that, in the case of chlorpyrifos, there are other factors which will also determine toxic impact (e.g. rate of metabolism, elimination rates, lipid content and body size; Rubach *et al.*, 2012). Nevertheless, this example shows that knowledge of fundamental physiological characteristics, and how these vary between organisms, can be informative in understanding and predicting toxicity.

This example is one of several studies that have shown a strong relationship between aquatic insect physiology and their sensitivity to chemical stressors. Correlations between aquatic insect biological traits and chemical exposure, have also been investigated for inorganic toxicants such as zinc (Buchwalter and Luoma, 2005), cadmium (Buchwalter *et al.*, 2008) and sulphate (Scheibener *et al.*, 2017).

## Maximizing mechanism

There is a growing understanding of the value of incorporating biological traits into risk assessment approaches. This is reflected in the growth of publications that feature mechanistic modelling considerations (Fig. 2). However, there is significant further scope for the incorporation of mechanistic knowledge into assessments of environmental risk. Below, selected examples of how physiological tools, frameworks, and data may contribute to a more robust understanding of the stressor-organism interaction are highlighted.

### Applying Krogh models

Among the founding doctrines of comparative physiology is Krogh’s principle, which states: ‘For a large number of problems there will be some animal of choice or a few such animals on which it can be most conveniently studied’ (Krogh, 1929). From a risk assessment perspective, Krogh’s principle could apply to those species that are most sensitive to a given stressor, as an NOEC established in the most sensitive species, will be protective of all. Water fleas (e.g. *Daphnia magna*) are often championed as excellent toxicological model species, owing in part to their high sensitivity to stressors (Baudo, 1987), and could therefore be considered a Krogh model. However, research utilizing a database of previous studies showed that, relative to *Daphnia magna*, 22% of taxa are more sensitive to organic toxicants, and 30% of taxa are more sensitive to trace metals (von der Ohe and Liess, 2004). This suggests that the most appropriate Krogh models may differ according to the stressors of relevance, and that identifying the most relevant toxicological Krogh model may not be a simple task (Cairns and Niederlehner, 1987; Rohr *et al.*, 2016).

However, physiological knowledge of the mechanisms by which stressors impact organismal health opens up new avenues for the application of Krogh’s principle. Such approaches might include the identification of environments that are particularly susceptible to stressor impact. For example, the finding that sodium ion turnover strongly correlates with silver ion toxicity (Wood, 2012), means that organisms inhabiting settings that already challenge sodium homoeostasis might be particularly at risk to silver toxicity. Such habitats would include waters of low pH, wherein high proton concentration inhibits sodium uptake, and forces these species to display extreme transport characteristics (Glover *et al.*, 2012). This could either make these environments home to particularly susceptible biota, or havens for species that have adaptations that would make them less sensitive to silver ion toxicity. Under either circumstance, these settings would be useful for identifying organisms most at risk, or for the identification of model species that would allow further elucidation of mechanisms of toxicity by identifying pathways that facilitate tolerance.

A more direct application of Krogh models to risk assessment is the study of animals capable of withstanding environments elevated in the stressor of interest. For example, populations of rainbowfish (*Melanotaenia nigrans*) have survived for several decades in copper-enriched waters associated with mining leachate in Australia. The tolerance of these fish appears to be related to their capacity to limit branchial copper uptake (Gale *et al.*, 2013). Characterization of the specific mechanisms involved could provide insight into factors that shape the sensitivity of species to copper, allowing a specific and refined risk assessment analysis approach in environments at risk of copper contamination. There are several other examples of settings where long-term exposure to a stressor has resulted in enhanced tolerance (Weis *et al.*, 1981; Uren Webster *et al.*, 2013; Lindberg *et al.*, 2017), but for very few of these instances is there a mechanistic understanding of how tolerance is achieved. These examples do, however, demonstrate that extremophile species and environments with extreme characteristics, offer promise for examining mechanisms of stressor action.

### Gathering mechanistic information from standard laboratory toxicity tests

While the weaknesses of standard toxicity tests are well recognized, they remain at the core of most risk assessment guidelines, and will likely remain so until new methodologies are embraced. There are, however, missed opportunities to gather mechanistic knowledge from these assessments. For example, simple measures of toxicant burden, can help link exposure to toxic effect (McCarty, 2015), and identify aspects of toxicant handling (e.g. distribution, metabolism, excretion) which can be informative of mechanisms of effect. Furthermore, adding measures of sub-lethal toxicity to standardized assays can help to identify mechanisms of impairment. These can be used to detect pathways of effect that are conserved between species and stressors, to identify unknown mechanisms of stressor action, and to allow prediction of sensitivity under non-standard exposure conditions. This is particularly true for tests conducted in novel species and/or with novel stressors, for which very little prior information may be available. A simple tool-box incorporating measurements of key end-points (e.g. bioaccumulation, metabolism, ion regulation, oxidative stress; see McRae *et al.*, 2018), could be an important addition to standard single end-point measurements in laboratory studies. Furthermore, such analyses are of significant value from an animal ethics perspective, in that they add data without increasing animal use.

### Delineating mechanisms of stressor action

Knowledge of how stressors cause adverse outcomes is essential for predicting sensitivity, and thus essential for proactive risk assessment. Recognizing this, some systems have been developed specifically for gathering mechanistic information related to stressor exposure. For example, an *in vivo* transected animal method has been previously employed to monitor up to 26 biochemical and physiological end-points in fish acutely exposed to stressors (McKim *et al.*, 1987). Although this technique has been largely superseded by the development of less invasive methods (Pettem *et al.*, 2017; Viant *et al.*, 2017), the underlying principle of collecting multiple end-points that will elucidate pathways of toxicant effect is one that has been widely adopted. Through these types of approaches, mechanisms of action are well understood for some stressors (e.g. ion mimicry for dissolved metals; impaired energy metabolism in hypoxia; conserved mechanisms of action of pharmaceuticals between target and non-target organisms, Brown *et al.*, 2014). However, for many groups of chemicals, there is surprisingly little understanding of toxic mechanism.

For organic chemical stressors, quantitative structure activity relationships (QSARs) have been used to deduce biological activity on the basis of shared structural similarities. However, for some of these chemical groupings, little is known regarding their mechanisms of action. For example, a large number of chemical stressors induce a narcotic effect, an ill-defined ‘baseline toxicity’ (Veith *et al.*, 1983), which is correlated with the hydrophobicity of the chemical toxicant (Escher and Hermens, 2002). This lack of mechanistic knowledge for narcotic chemicals hinders prediction of organism responses to these chemicals, both alone and as components of complex mixtures (Escher and Hermens, 2002). However, recent work has suggested that these toxicants may have a common mechanism of impairment. Exposure of *Daphnia magna* to chemicals that induce narcosis, resulted in transcriptional profiles over-enriched in genes associated with calcium homoeostasis (Antczak *et al.*, 2015). The magnitude of transcriptional change correlated to the hydrophobicity of the chemical, which in turn was related to the capacity of the chemical to specifically inhibit components of intracellular calcium homoeostasis (i.e. the sarco/endoplasmic reticulum calcium ATPase). Importantly, alterations in calcium handling were then able to be related to changes in *Daphnia* heart rate (Antczak *et al.*, 2015), an easily monitored physiological end-point. This AOP provides valuable mechanistic information of relevance to risk assessment, and is thus an exemplar of experimental approaches that are required to enhance the mechanistic basis of risk assessments.

### Field-based physiological techniques

There are a number of practical reasons why risk assessments are restricted to a small subset of organisms that are amenable to laboratory culture. Laboratory-reared animals have a known and constant exposure history and, as they are often inbred, have a reduced genetic diversity that minimizes variability in responses to toxicants (Ballatori and Villalobos, 2002; Segner and Baumann, 2015). However, laboratory models are not always ecologically relevant, and their responses may be poorly representative of other members of their taxonomic group (Cairns and Niederlehner, 1987).

There are a number of physiological end-points (e.g. metabolic rate, and in aquatic settings, ion regulation), that seem particularly valuable for assessing the risks posed by environmental stressors. These are also parameters that can be measured in the field, using non-lethal techniques (Wood *et al.*, 2002; Butler *et al.*, 2004; Mochnacz *et al.*, 2017). Consequently, there is capacity to conduct simple physiological measurements on organisms directly sampled from areas of specific interest. Such areas could include sites of particular ecological value, and/or sites considered particularly at risk or already impacted by stressors. Field-collected data could therefore provide fundamental information regarding the physiology of species of greatest relevance to field settings, under natural exposure conditions. Combined with mechanistic knowledge of stressor action, this information may be used to proactively highlight sensitive species (i.e. species with extreme rates of basal functions targeted by stressors). In areas that are already impacted by stressors, comparisons with control sites could identify species traits that have led to extirpation (White *et al.*, 2017), elucidating unknown mechanisms of stressor effect. Although there are a number of important practical considerations, including confounding effects associated with exposure history and handling (Harley and Glover, 2014), this general approach of applying field-based physiological methods to directly interrogate regions of interest has significant value. It facilitates the integration of individual-level measurements with ecological relevance, a key pitfall of current risk assessment approaches (Rohr *et al.*, 2016).

## Conclusions

Current regulatory practices focus on deriving simple toxicological metrics, and as such do not consider the biological mechanisms driving toxicity (Jager, 2016). Furthermore, extrapolation of laboratory tests in model organisms to effects on biota in ecosystems does not account for the often distinct physiology of non-model species, which will alter the toxicological impact of the stressor. Knowledge of how stressors cause their effects facilitates predictive modelling by accounting for species differences and the influence of environmental variables that impact biological function. Mechanistic information is also key for approaches assessing the impact of multiple stressors (Segner *et al.*, 2014). Critically, predicting stressor sensitivity on the basis of molecular, biochemical and physiological traits minimizes the need for extensive lethal toxicity testing. The identification of conserved physiological characteristics that underlie sensitivity to a given stressor will facilitate cross-species extrapolation without having to utilize large number of animals (as in the case of LC_50_ assessments), and which can be performed in the absence of the stressor (e.g. sodium uptake characteristics as a determinant of silver toxicity). This will have significant benefits in terms of cost and ethical considerations (Burden *et al.*, 2015).

There are, however, significant challenges to integrating physiological measures into risk assessment approaches. As for toxicological studies, research on organismal physiology is often focussed on a few model species, and thus there is often only limited knowledge of physiological function in the most relevant species to an environment of concern. Model parametrization will rely absolutely on expanding fundamental knowledge, and this is therefore a major obstacle to adopting effective mechanism-based approaches. Similarly, attaining the weight of evidence needed to link physiological changes to toxicity is challenging (Hahn, 2011). Consequently, for many stressors there is still significant research effort required to establish reliable pathways of effect. Finally, the complexity of environmental exposure scenarios is a major hurdle for the development of effective traits-based risk prediction. The spatial and temporal variation in stressor magnitude, the myriad of complicating environmental factors, and the capacity of the organism to acclimate to stressor exposure will all compromise effective prediction. Nevertheless, the successful implementation of physiology-based models such as the BLM (Rüdel *et al.*, 2015), show that these challenges can be largely overcome, and that there is significant scope for, and value in, considering organism physiology in risk assessment approaches.
